# Characterization of the Therapeutic Profile of Albiflorin for the Metabolic Syndrome

**DOI:** 10.3389/fphar.2019.01151

**Published:** 2019-10-11

**Authors:** Xiu Zhou, Sherouk Fouda, Xiao-Yi Zeng, Dongli Li, Kun Zhang, Jun Xu, Ji-Ming Ye

**Affiliations:** ^1^School of Biotechnology and Health Sciences, Wuyi University, Jiangmen, China; ^2^School of Health and Biomedical Sciences, RMIT University, Melbourne, VIC, Australia; ^3^School of Biomedical and Pharmaceutical Sciences, Guangdong University of Technology, Guangzhou, China

**Keywords:** albiflorin, metabolic syndrome, type 2 diabetes, non-alcoholic steatohepatitis, energy expenditure, physical activity

## Abstract

Albiflorin (AF) is a small molecule (MW 481) isolated from *Paeoniae radix*, a plant used as a remedy for various conditions with pathogenesis shared by metabolic diseases. Reported here is our characterization of its therapeutic profiles in three mouse models with distinctive pathological features of metabolic syndrome (MetS). Our results firstly showed that AF alleviated high fat (HF) induced obesity and associated glucose intolerance, suggesting its therapeutic efficacy for MetS. In the type 2 diabetes (T2D) model induced by a combination of HF and low doses of streptozotocin, AF lowered hyperglycaemia and improved insulin-stimulated glucose disposal. In the non-alcoholic steatohepatitis-like model resulting from a HF and high cholesterol (HF-HC) diet, AF reversed the increased liver triglyceride and cholesterol, plasma aspartate aminotransferase, and liver TNFα mRNA levels. Consistent with its effect in promoting glucose disposal in HF-fed mice, AF stimulated glucose uptake and GLUT4 translocation to the plasma membrane in L6 myotubes. However, these effects were unlikely to be associated with activation of insulin, AMPK, ER, or cellular stress signalling cascades. Further studies revealed that AF increased the whole-body energy expenditure and physical activity. Taken together, our findings indicate that AF exerts a therapeutic potential for MetS and related diseases possibly by promoting physical activity associated whole-body energy expenditure and glucose uptake in muscle. These effects are possibly mediated by a new mechanism distinct from other therapeutics derived from Chinese medicine.

## Introduction

Metabolic syndrome (MetS) is a cluster of closely related metabolic disorders including central obesity, insulin resistance, hyperglycaemia, dyslipidaemia, and associated various manifestations in different organs (Eckel et al., 2010). One resultant metabolic disease is type 2 diabetes (T2D), which has become a major non-communicable disease worldwide. The increasing prevalence of diabetes results in an enormous health and social burden (Wild et al., 2004). MetS is also a risk factor to multiple organs, among which non-alcoholic fatty liver disease (NAFLD) is increasingly recognized as a manifestation of MetS in the liver (Diehl and Day, 2017). While associated with T2D, NAFLD alone presents as another serious disease affecting 20–30% adult population (Younossi et al., 2018) and it usually starts from an excess accumulation of triglycerides. Approximately, 8–20% of hepatosteatosis progresses to non-alcoholic steatohepatitis (NASH) with inflammation, injury, and even fibrosis in the liver. The latter may further advance to irreversible cirrhosis or even carcinoma (Diehl and Day, 2017; Younossi et al., 2018). As the comorbidities of MetS are complicated and heterogenous among populations, it is important to investigate the therapeutical profiles of an anti-MetS drug suitable for multifaceted pathological features of MetS.

In search for possible new therapeutics for MetS derived conditions, we have taken an approach of drug repurposing to target traditional Chinese medicine (TCM) (Turner et al., 2016). This approach has been proven to be fruitful in identifying novel classes of compounds with promising anti-diabetic properties (Tan et al., 2008; Turner et al., 2008; Zeng et al., 2015). Albiflorin (AF) is a small molecule (MW 481) containing a unique cage-like pinane skeleton. It is rich in herbal remedies such as *Paeoniae radix* (Morinaga et al., 2013) with a good oral absorption property and pharmacokinetics *in vivo* (Li et al., 2011). Here we have selected AF for the present study based on the reported implications relevant to MetS and associated conditions. Firstly, some AF derivatives have been reported to lower hyperglycaemia in diabetic animals independent of insulin secretion (Hsu et al., 1997; Juan et  al., 2010) and hyperlipidaemia (Yang et al., 2004). One of our previous studies showed that AF can reduce the accumulation of intracellular lipids in cultured 3T3-L1 adipocytes (Zeng et al., 2012b). Additionally, AF has also been shown to protect against kidney injury in diabetic animals (Zhang et al., 2009). However, the therapeutical profiles of AF for MetS and associated comorbidities have not been well characterized.

Therefore, the present study aims to examine the efficacy of AF for MetS and associated comorbidities resembled in three nutrient-based mouse models which have been well characterized in our laboratory. These models included 1) obesity and associated insulin resistance resulting from a high fat (HF) diet (Turner et al., 2007; Tan et al., 2008), 2) a T2D model induced by HF diet and low doses of streptozotocin (STZ) (Zeng et al., 2012a) to evaluate its therapeutic properties for hyperglycaemia, and 3) a NAFLD/NASH model generated by HF and high cholesterol (HF-HC) diet (Savard et al., 2013; Li et al., 2016) to further examine its efficacy for the manifestation of MetS in the liver. The rationale for these studies is that these three nutrient-based animal models would provide a wide spectrum of major pathological phenotypes of MetS to allow proper evaluation of the therapeutic profiles of AF for MetS and their related disorders.

## Materials and Methods

### Compound

**Figure 1:**
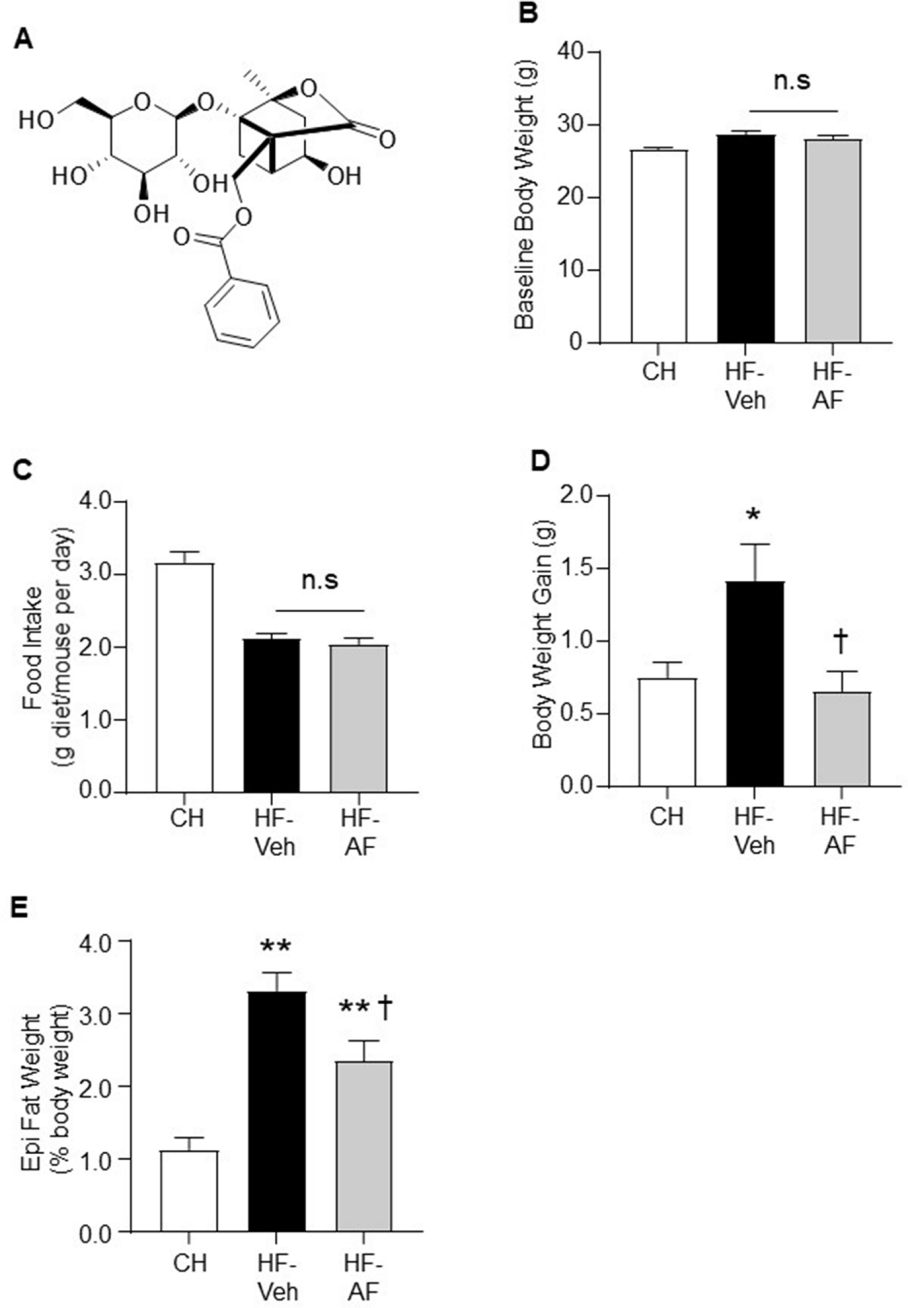
Effects of AF on body weight gain and epididymal fat mass in HF-fed mice. **(A)** Structure of albiflorin (AF). AF is a monoterpene glucoside containing a unique cage-like pinnae skeleton. C57BL/6J mice were fed a high fat (HF) diet for 8 weeks and AF (100 mg/kg/day) was added to the HF diet for the last 2 weeks, body weight and food intake were monitored on a weekly basis. Food intake was calculated as an average daily intake per mouse of the same group over the last 2 weeks. **(B)** Baseline body weight prior to AF administration, **(C)** Food intake and **(D)** Body weight gain during the last 2 weeks, and **(E)** Epididymal (Epi) fat weight at the end of the experiment. CH - chow-fed normal mice, HF-Veh - untreated high fat fed mice, HF-AF - high fat fed mice treated with AF. Data are shown as means ± SEM of 7-10 mice for each group. n.s, no significant differences; *p < 0.05, **p < 0.01 vs CH; ^†^p < 0.05 vs HF-Veh.

AF (**Figure 1A**) was prepared by silica gel column chromatography (eluted with ethyl acetate saturated with water) from commercial total glycosides of *Paeoniae* (Xian Helin Biological Engineering Co., Ltd., Shanxi, China). The structures of AF was determined by comparing NMR spectroscopic data with published values (Yamaski et al., 1976; Lemmich, 1996) and the purity (>98%) was verified by HPLC analysis.

### Animals

Male C57BL/6J mice (8–10 weeks old) were purchased from the Animal Resources Centre (Perth, Australia). The mice were kept at 22 ± 1°C on a 12-h light/dark cycle with free access to food and water. All interventions were performed on separate groups of animals (n = 7–10 mice for each group unless indicated elsewhere). Body weight and food intake were monitored over all groups on a weekly basis.

### Animal Models of MetS With Different Features

The effect of AF on MetS was first examined in a widely used HF diet induced insulin resistant mouse model which develops obesity and glucose intolerance (Turner et al., 2007; Tan et al., 2008). After one-week acclimatisation, mice were fed a HF diet (45% lard) for 8 weeks with or without AF (100 mg/kg/day) (Xu et al., 2011) in the HF diet for the final 2 weeks. Since HF-mice are known to lack significant hyperglycaemia to represent overt T2D, we next induced a MetS associated T2D by HF feeding and low doses of STZ to examine the effect of AF on the hyperglycaemia in this T2D model. Briefly, mice were fed a HF diet for 12 weeks (to produce insulin resistance) in combination with low doses of STZ (50 mg/kg, intraperitoneal injection for 5 consecutive days) to disable the compensatory increase in insulin production (Zeng et al., 2012a). Half mice in HF-STZ group were fed with AF (100 mg/kg/day in HF diet) for the final 3 weeks. Recent studies from us and other groups have shown that addition of cholesterol in HF diet produces a NAFLD/NASH-like model with MetS characteristics (Savard et al., 2013; Li et al., 2016). Therefore, we further examine the effect of AF in this model, which represents a manifestation of the MetS in the liver. In this experiment, mice were fed HF-HC diet for 9 weeks to generate a MetS model with NAFLD/NASH-like phenotype treated with AF (100 mg/kg/day in HF-HC diet) in the last 4 weeks.

### Measurements of Blood and Plasma Parameters

Intraperitoneal glucose tolerance test (ipGTT, glucose load 2.0 g/kg body weight) and insulin tolerance test (ipITT, 1 U/kg insulin, Actrapid, Novo Nordisk) were performed after 5–7 h fasting. Blood samples were collected from the tail tip and blood glucose levels were monitored at designated time points using an Accu-Chek glucometer (Roche Diagnostics, Australia).

After centrifugation, plasma was transferred to a new Eppendorf tube and stored at -80°C for subsequent measurement.Plasma aspartate aminotransferase (AST) was determined by a commercial enzymatic kit (Laboratory Diagnostics Pty. Ltd., #ST2920-500). Briefly, working reagent was added to each plasma sample and incubated at 37°C. Absorbance at 340 nm was recorded every 1 min for 10 min using a Polarstar Optima microplate reader (BMG Lab Technologies, Germany). Distilled water was used as a blank.

### Determination of Tissue Triglyceride and Cholesterol Levels

Mice were sacrificed by cervical dislocation and liver samples were freeze-clamped immediately and stored at -80°C for subsequent analysis. After extracting lipids by Bligh and Dyer’s method (Bligh and Dyer, 1959), liver triglyceride and cholesterol levels were measured using a Triglyceride GPO-PAP kit (Roche Diagnostics, Australia) and a Cholesterol CHOD-PAP kit (Roche Diagnostics, Australia), respectively. The absorbance was determined spectrophotometrically using a Polarstar Optima microplate reader (BMG Lab Technologies, Germany) as described in the previous publications from this laboratory (Ren et al., 2012; Chan et al., 2013).

### Histological Analysis

At the end of the experiment, fresh liver samples from each group were placed in a tissue embedding cassette and fixed in 10% neutral-buffered formalin solution at 4°C overnight. The next day, samples were transferred to 70% ethanol and processed with a tissue processor (Leica, Wetzlar, Germany). The processed liver samples were then embedded in paraffin and cut into 5 µm thickness sections followed by hematoxylin and eosin (H&E) staining for microscopic examination. Images (five from each section) were captured randomly at 20x magnification and scored in a double-blind manner. Hepatocyte ballooning was quantified as described by Kleiner et al. (Kleiner et al., 2005) and the average score of all the samples from the same group was reported.

### Quantitative Real-Time PCR

Total RNA from freeze-clamped liver samples were extracted with TRIZOL (Invitrogen, USA) according to the manufacturer’s instructions. Real-time PCR for tumour necrosis factor alpha (TNFα) (Genework, Australia) was performed using the IQ SYBR Green Supermix. Gene expression for each sample was analysed in duplicates and normalised to the housekeeping gene 18S. The primer sequences (5’ to 3’) of 18S were: CGCCGCTAGAGGTGAAATTCT (sense) and CGAACCTCCGACTTTCGTTCT (antisense); TNFα: CACAAGATGCTGGGACAGTGA (sense) and TCCTTGATGG TGGTGCATGA (antisense). All reactions were performed on the iQ^™^ 5 Real-time PCR Detection System (Bio-Rad Laboratories Inc., USA) as previous publications from this laboratory (Ye et al., 2011; Zeng et al., 2012a).

### Studies in L6 Myoblasts

L6 myoblasts (up to passage 15) were cultured in α-minimal essential medium (α-MEM, Gibco) supplemented with 10% heat-inactivated foetal bovine serum (FBS, Gibco) and 1% antibiotic/antimycotic (Gibco) at 37°C in a humidified 5% CO_2_ incubator. For the differentiation of myotubes, cells were cultured in α-MEM supplemented with 2% heat-inactivated FBS and 1% antibiotic/antimycotic at 37°C with 5% CO_2_ for 5–7 days before interventions.

#### Glucose Uptake Assay

The effect of AF on glucose uptake was examined using 2-deoxy-D [2,6-3H] glucose based on previous publication (Qiu et al., 2010). Briefly, differentiated L6 myotubes were serum starved for 18 h in α-MEM containing 4% BSA. AF (10 μM) was included for the final 1 h before being quickly washed off. Cells were then maintained in PBS containing 1 mM CaCl_2_, 1 mM MgCl_2_, 1 mM MgSO_4,_ and 0.2% BSA, with or without addition of insulin (100 nM) for 20 min. For the final 5 min, 10 µmol/l 2-deoxy-D [2,6-^3^H] glucose (200 nCi/ml) (Amersham Biosciences, Buckinghamshire, U.K.) was added. Medium was quickly removed and cells were immersed in ice-cold PBS before being lysed in lysis buffer (50 mM HEPES pH 7.4, 150 mM NaCl, 1% Triton X-100, 1 mM EDTA, 10% glycerol) containing phosphatase inhibitors (20 mM NaF, 2 mM Na_4_P_2_O_7_, 2 mM Na_3_VO_4_). Radioactivity was determined by liquid scintillation counting and protein content was determined by the BCA method (Pierce, Rockford, IL, USA).

#### GLUT4 Translocation Assay

To assess GLUT4 translocation to the plasma membrane, L6 myoblasts were infected with replication-incompetent retroviruses expressing HA-GLUT4 and selected with puromycin before differentiation into myotubes as described previously (Lee et al., 2006). Cells were then subjected to 10 μM AF in PBS for 1 h, followed by either vehicle or 100 nM insulin treatment for 30 min. The HA-GLUT4 translocation assay was then performed as per previous (Hoehn et al., 2008).

#### Western Blotting

The insulin, AMPK and stress pathways in L6 myotubes, were assessed by western blotting for the key proteins as described before (Zeng et al., 2015). Briefly, L6 myotubes were serum starved in α-MEM for a total of 18 h before being quickly washed twice on ice-cold PBS. Cells were lysed in lysis buffer supplemented with Complete Protease Inhibitor mixture (Roche, Mannheim, Germany) on ice and then freeze-thawed in liquid nitrogen. Cell debris was removed by centrifugation at 4°C and protein content was quantified by BCA protein assay. Laemmli’s buffer containing 15 mM DTT was added to an aliquot of tissue lysate and incubated at 65°C for 10 min. Lysates were then subjected to SDS-PAGE on 9% mini gels and transferred onto Hybond PVDF membranes (GE Healthcare, Buckinghamshire, UK) before immunoblotting. The phospho (Tyr612, #sc17195) and total (#sc8038) IRS-1 antibodies were from Santa Cruz (USA). The phospho (Ser724, #ab48187) and total (#ab37073) IRE-1 antibodies were from Abcam (UK). The phospho (Ser473, #9271; Thr308, #9275) and total Akt (#9272), phospho (Ser9, #9323) and total (#9315) GSK3β, phospho p38 MAPK (Thr180/Tyr182, #9211) and total (#9212) p38 MAPK, phospho (Thr202/Tyr204, #4370) and total (#4695) ERK, phospho (Ser485, #4184; Thr172, #2535) and total (#2532) AMPKα, phospho (Ser79, #3661) and total (#3662) ACC, phospho (Thr642, #8881) and total (#2670) AS160, phospho (Ser51, #3597) and total (#2103) eIF2α, phospho (Thr183/Tyr185, #9251) and total (#9252) JNKα/β, and phospho (Ser32, #2859) and total (#9242) IκBα antibodies were from Cell Signaling Technology (USA).

### Measurement of Whole-Body Metabolic Rate and Physical Activity

To measure the acute effect of AF on whole body metabolic rate, an indirect calorimeter (Oxymax, Columbus Instruments, USA) was used as described previously (Tan et al., 2008). Briefly, CH-fed mice (12-week old) were first acclimatized in the Oxymax system for 2 h. The volume of O_2_ consumption (VO_2_) and total activities were continuously monitored for 21 h after mice received vehicle (0.5% methylcellulose) or AF (100 mg/kg body weight, dissolved in 0.5% methylcellulose) by oral gavage.

### Statistical Analysis

Data are presented as means ± SEM. One-way ANOVA was used to determine the statistical significance across three groups. When significant differences were found, Tukey’s multiple comparisons tests were used to examine differences between groups. Unpaired t-test was used for comparison between two groups. Data analysis was performed using GraphPad Prism software (8.0, GraphPad Software Inc, CA, USA). A difference of p < 0.05 is considered to be statistically significant.

## Results

### Effects of AF on Body Weight Gain and Epididymal Fat Mass in HF-Fed Mice

Firstly, the study was conducted in an insulin resistant mouse model induced by HF diet feeding, in which AF was administrated to the HF mice for the last 2 weeks. The baseline body weight of the untreated HF-fed (HF-Veh) and HF-fed treated with AF (HF-AF) groups were similar prior to the AF administration (**Figure 1B**). Oral administration of AF had no effect on the amount of intake of the HF diet (p > 0.05 between HF-Veh and HF-AF, **Figure 1C**). Compared to CH-fed mice (CH group), HF-Veh mice displayed ∼2-fold greater weight gain (**Figure 1D**) and ∼3-fold increase in epididymal fat pad (**Figure 1E**). However, the body weight gain of HF-AF mice was significantly reduced (p < 0.05 vs HF-Veh) and remained at a level similar to CH-fed mice (**Figure 1D**). Similarly, AF treatment significantly reduced the epididymal fat pad in HF-fed mice (**Figure 1E**).

### Effects of AF on Glucose Intolerance in HF-Fed Obese Insulin Resistant Mice

**Figure 2:**
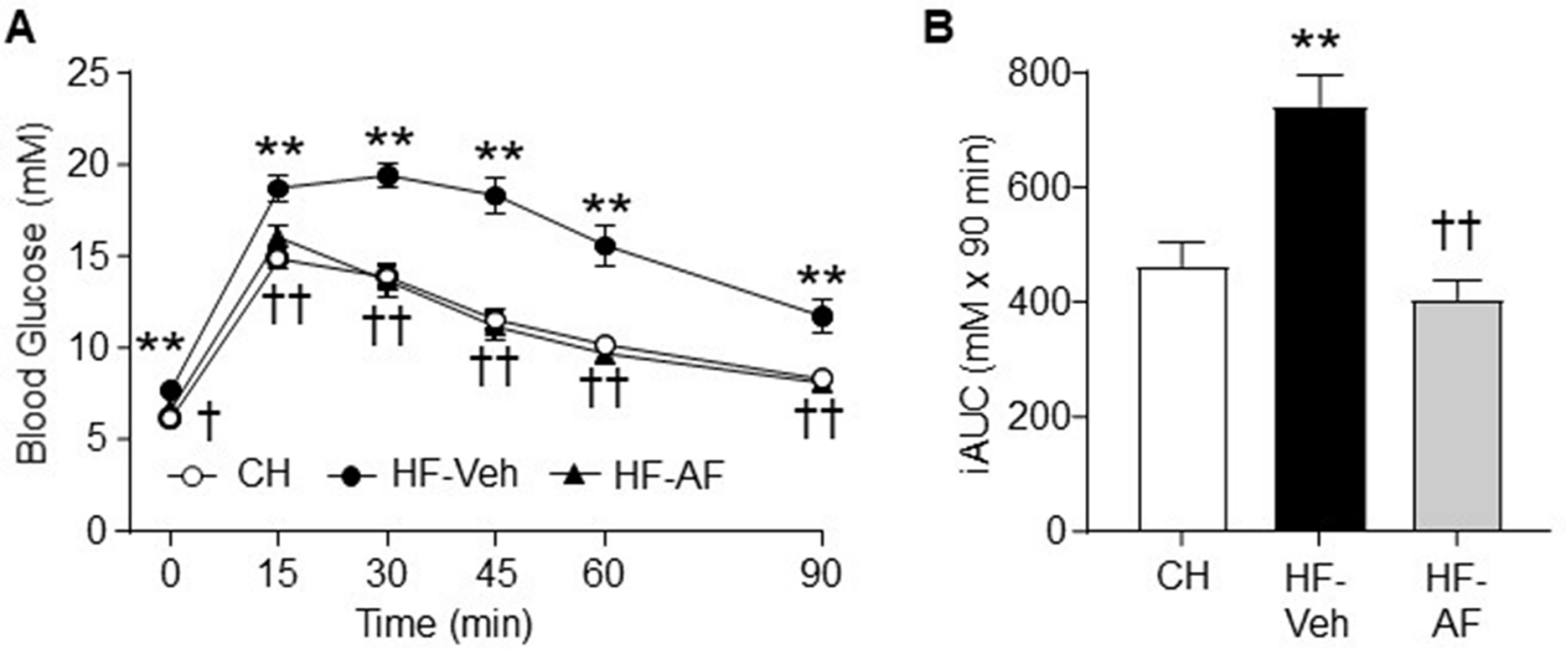
Effects of AF on glucose intolerance in HF-fed obese insulin resistant mice. Glucose tolerance test (GTT, 2.0 g glucose/kg body weight, *i.p*.) was performed after 2 weeks of AF treatment. **(A)** Blood glucose excursion over time, **(B)** Incremental area under the curve (iAUC) of blood glucose levels during the GTT. CH - chow-fed normal mice, white circle; HF-Veh - untreated high fat fed mice, black circle; HF-AF - high fat fed mice treated with AF, black triangle. Data are shown as means ± SEM of 7-8 mice for each group. **p < 0.01 vs. CH; ^†^p < 0.05, ^††^p < 0.01 vs. HF-Veh.

To assess the effect of AF on glucose homeostasis, mice were subjected to an ipGTT. As shown in **Figure 2A**, as expected, HF-Veh mice showed significant higher levels of blood glucose throughout the period of the ipGTT compared to CH-fed mice. Compared to CH-fed mice, the calculated incremental area (iAUC) during the ipGTT of HF-Veh mice was increased by 60% (p < 0.01). Administrated of AF (HF-AF) restored glucose tolerance to a level almost identical to that of the CH-fed mice (**Figures 2A, B**). Together these data suggest that AF can attenuation some aspects of the diet-induced insulin resistance through an improved glucose disposal.

### Effects of AF on Hyperglycaemia and Insulin Intolerance in Diabetic Mice Induced by HF Diet and Low Doses of STZ

**Figure 3:**
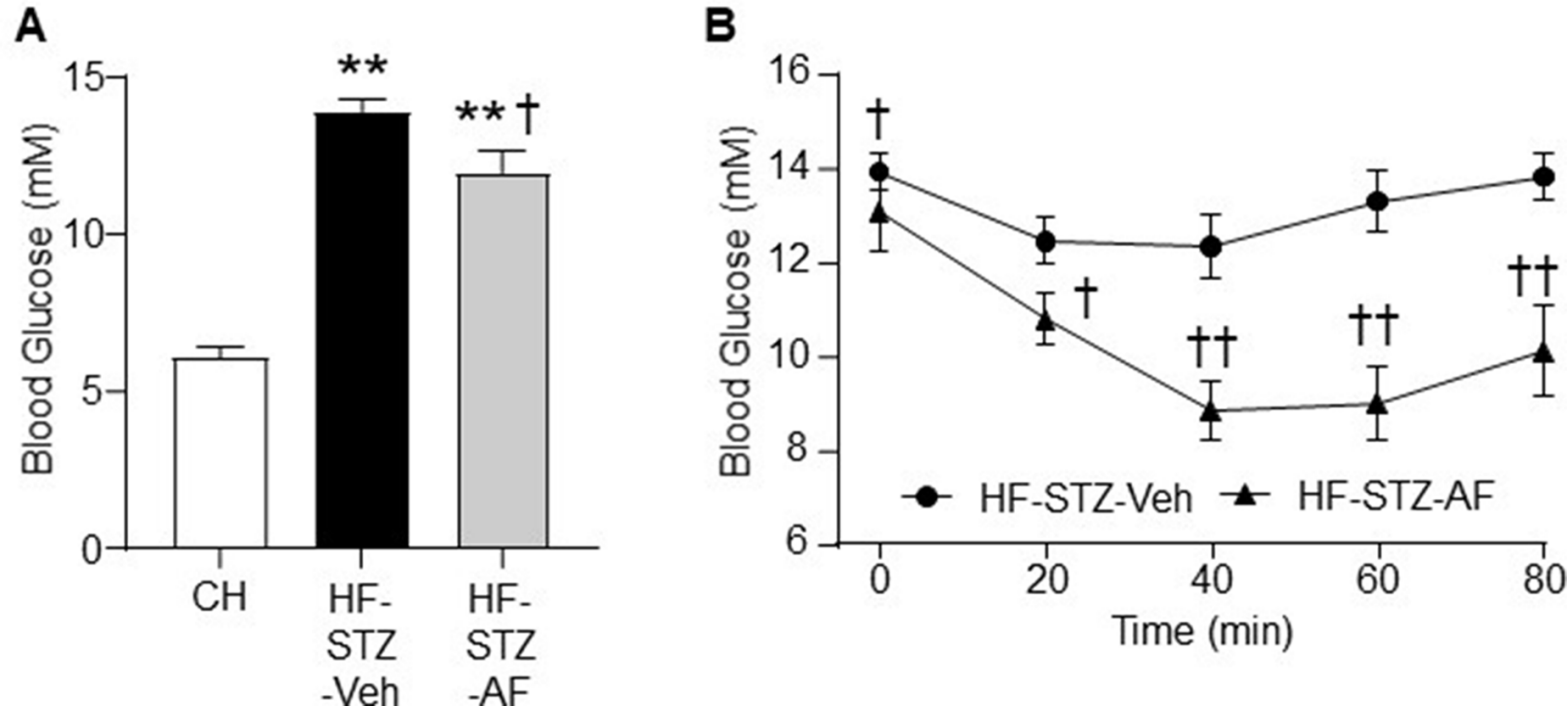
Effects of AF on hyperglycaemia and insulin intolerance in diabetic mice induced by HF diet and low doses of STZ. The mouse model of type 2 diabetes (T2D) was generated by HF diet (to induce insulin resistance) for 12 weeks and injections (*i.p*) of streptozotocin (STZ) at a low dose (50 mg/kg) for the first 5 days of the diet (to block the compensatory increase in insulin secretion for HF-induced insulin resistance). Once the hyperglycaemia (T2D) was stabilised, AF (100 mg/kg/day) was added to the HF diet for 3 weeks. **(A)** Fasting blood glucose after 2 weeks of AF treatment and **(B)** Insulin tolerance test (ITT, insulin 1.0 IU/kg body weight *i.p*. injection) in untreated diabetic mice (HF-STZ-Veh, black circle) and treated diabetic mice (HF-STZ-AF, black triangle) at the end of the study. Data are shown as means ± SEM of 6-8 mice for each group. **p < 0.01 vs CH; ^†^p < 0.05, ^††^p < 0.01 vs. HF-STZ-Veh.

As overt T2D is characterised by hyperglycaemia due to peripheral insulin resistance together with impaired pancreatic β-cell function, we assessed the effect of AF on insulin sensitivity in a STZ-induced hyperglycaemic animal model. Mice fed a HF diet for 12 weeks with multiple low dose STZ injections for the first 5 days of the diet (HF-STZ-Veh) demonstrated a marked hyperglycaemia (by 2.2-fold, p < 0.01 vs CH) (**Figure 3A**). The presence of AF in the HF for 2 weeks (HF-STZ-AF) significantly reduced the hyperglycaemia (p < 0.05 vs HF-Veh) (**Figure 3A**). To assess the peripheral insulin sensitivity, we subjected these mice to an ipITT. Hyperglycaemic mice treated with AF (HF-STZ-AF) had a significantly improved response to exogenous insulin by ipITT, suggesting an improved peripheral insulin sensitivity (**Figure 3B**).

### Effects of AF on NAFLD/NASH Mice Induced by HF-HC Diet

**Figure 4:**
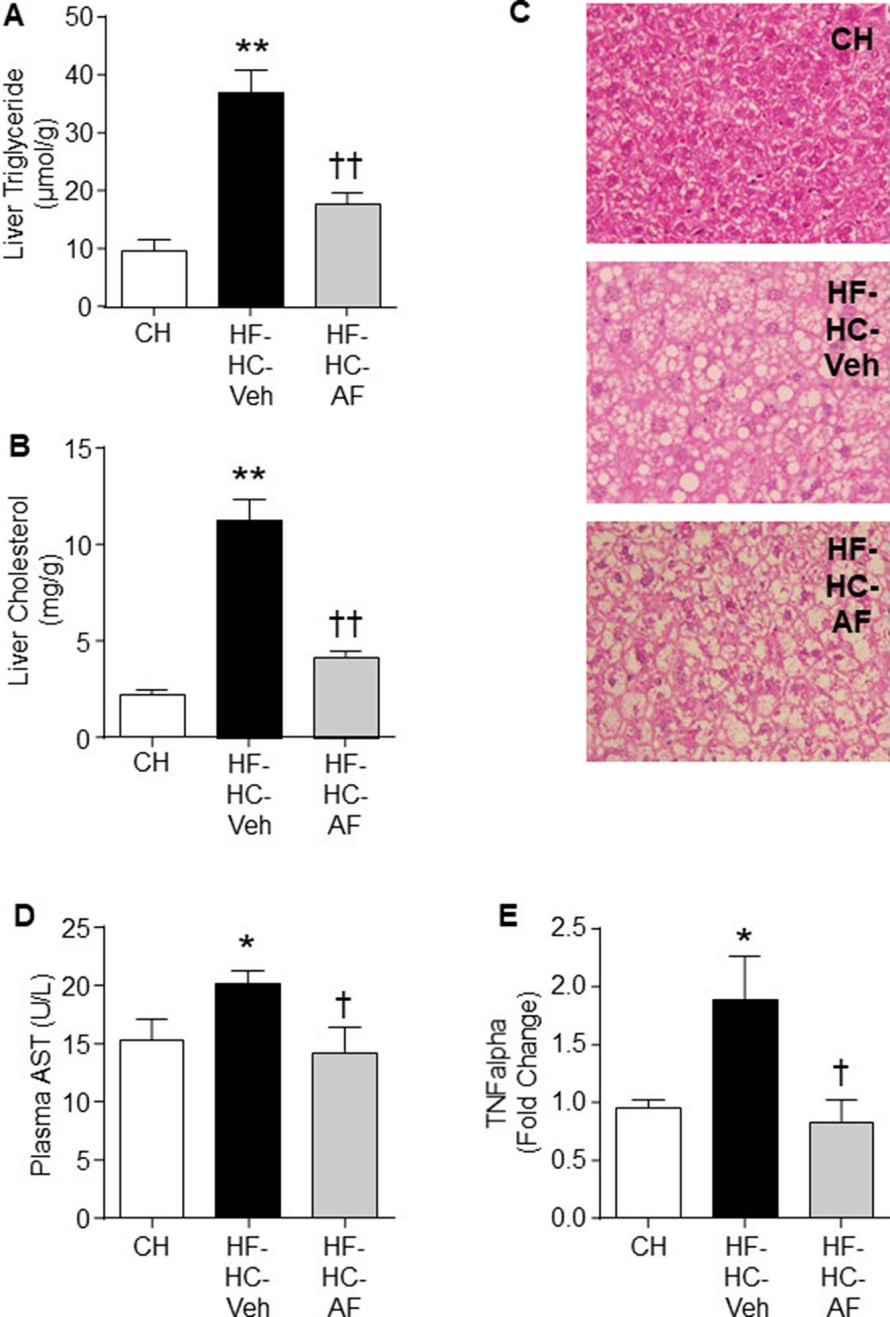
Effects of AF on NAFLD/NASH mice induced by HF-HC diet. C57BL/6J mice were fed a CH diet or HF-HC diet for 13 weeks and AF (100mg/kg/day in HF-HC diet, HF-HC-AF group) was given for the last 4 weeks. Mice were sacrificed by cervical dislocation. Plasma samples were collected and liver samples were freeze-clamped immediately and stored in -80°C. Liver **(A)** Triglyceride and **(B)** Cholesterol levels, and **(C)** Representative images (at 20x) of the H & E staining. Vacuoles indicate the pattern and shapes of lipid accumulation. **(D)** Plasma level of AST (an enzyme indicative of liver injury) and **(E)** TNFα mRNA expression in the liver. The expression levels were determined by quantitative real-time PCR and normalized by 18S mRNA. Quantified data are expressed as means ± SEM of 7-14 mice for each group. *p < 0.05, **p < 0.01 CH; ^†^p < 0.05, ^††^p < 0.01 vs. HF-HC-Veh.

We further tested the efficacy of AF in a NAFLD/NASH-like model with the manifestation of MetS. Insulin resistance is strongly associated with the ectopic lipid accumulation in insulin-target tissues (Samuel and Shulman, 2012). Therefore, we determined the levels of triglyceride and cholesterol to investigate the effects of AF on lipid accumulation in the liver, which is a major organ response to insulin action. As expected, HF-HC-fed mice (HF-HC-Veh) showed a significant increase in levels of liver triglyceride (by 3.9-fold) and cholesterol (by 5-fold) (both p < 0.01 vs CH). AF administration for 4 weeks significantly reduced the liver triglyceride (by 52%) and cholesterol (by 63%) levels in HF-HC fed mice (HF-HC-AF group, p < 0.01) (**Figures 4A, B**).

People with benign hepatic steatosis may develop to more severe steatohepatitis (Cohen et al., 2011). In the present study, H&E staining was performed and hepatocyte ballooning was used as diagnostic criteria to distinguish between steatohepatitis and simple steatosis (Kleiner et al., 2005). The HF-HC group presented a dramatic increase in hepatocellular swollen and rarefied cytoplasm (ballooning, 8.6 ± 4.3 vs 0.0 ± 0.0 in CH group, p < 0.01). Four-week administration of AF did not improve the severe hepatocyte ballooning in HF-HC-fed mice (8.9 ± 4.1 vs 8.6 ± 4.0 in HF-HC group, p > 0.05) (**Figure 4C**).

Plasma AST is commonly used as a marker to indicate liver injury. HF-HC mice presented higher AST level than the CH-fed mice, while AF significantly decreased the AST level in HF-HC-fed mice (both p < 0.05, **Figure 4D**). AF has shown several beneficial effects on inflammatory conditions (Chang et al., 2009; Kim and Ha, 2010; Jiang et al., 2011). Therefore, we next measured the gene expression of liver pro-inflammatory cytokine TNFα to determine whether AF-mediated reduction of hepatic lipid accumulation may lead to reduced inflammation. The HF-HC group showed a marked evaluation of TNFα gene expression by ∼40% (p < 0.05 vs CH), while AF treatment significantly restored the TNFα expression level in the liver of HF-HC mice (p < 0.05 vs HF-HC-Veh) (**Figure 4E**).

Inﬂammation is closely related to insulin resistance, obesity, and T2D (Diehl and Day, 2017; Younossi et al., 2018). As AF was shown to attenuate the gene expression level of pro-inflammatory cytokine TNFα in the liver, the effects of AF on glucose metabolism were then examined. As expected, HF-HC-fed mice showed significant increases in fasting blood glucose (by 46%, p < 0.01) compared to CH-fed mice. AF significantly lowered the fasting blood glucose to the level of the CH mice (**Table 1**).

**Table 1:** Effects of AF on blood glucose levels in NAFLD/NASH mice induced by HF-HC diet.

		CH	HF-HC-Veh	HF-HC-AF
**Fasting glucose (mM)**	Prior to AF treatment	10.1 ± 0.3	10.6 ± 0.3	11.3 ± 0.5
	After AF treatment	8.7 ± 0.6	12.8 ± 0.8**	9.4 ± 0.5^††^

Fasting blood glucose level for C57BL/6J mice fed 13 weeks on either a standard chow (CH) diet or a high fat high cholesterol diet (HF-HC) in the absence (HF-HC-Veh) or presence of albiflorin (AF, 100 mg/kg/day) (HF-HC-AF) for the last 4 weeks. Fasting blood glucose was assessed before and after AF administration. Data are shown as means *±* SEM of 7-8 mice for each group. ** p < 0.01 vs. CH; ^††^p < 0.01 vs. HF-HC-Veh.

### Effects of AF in L6 Myotubes

**Figure 5:**
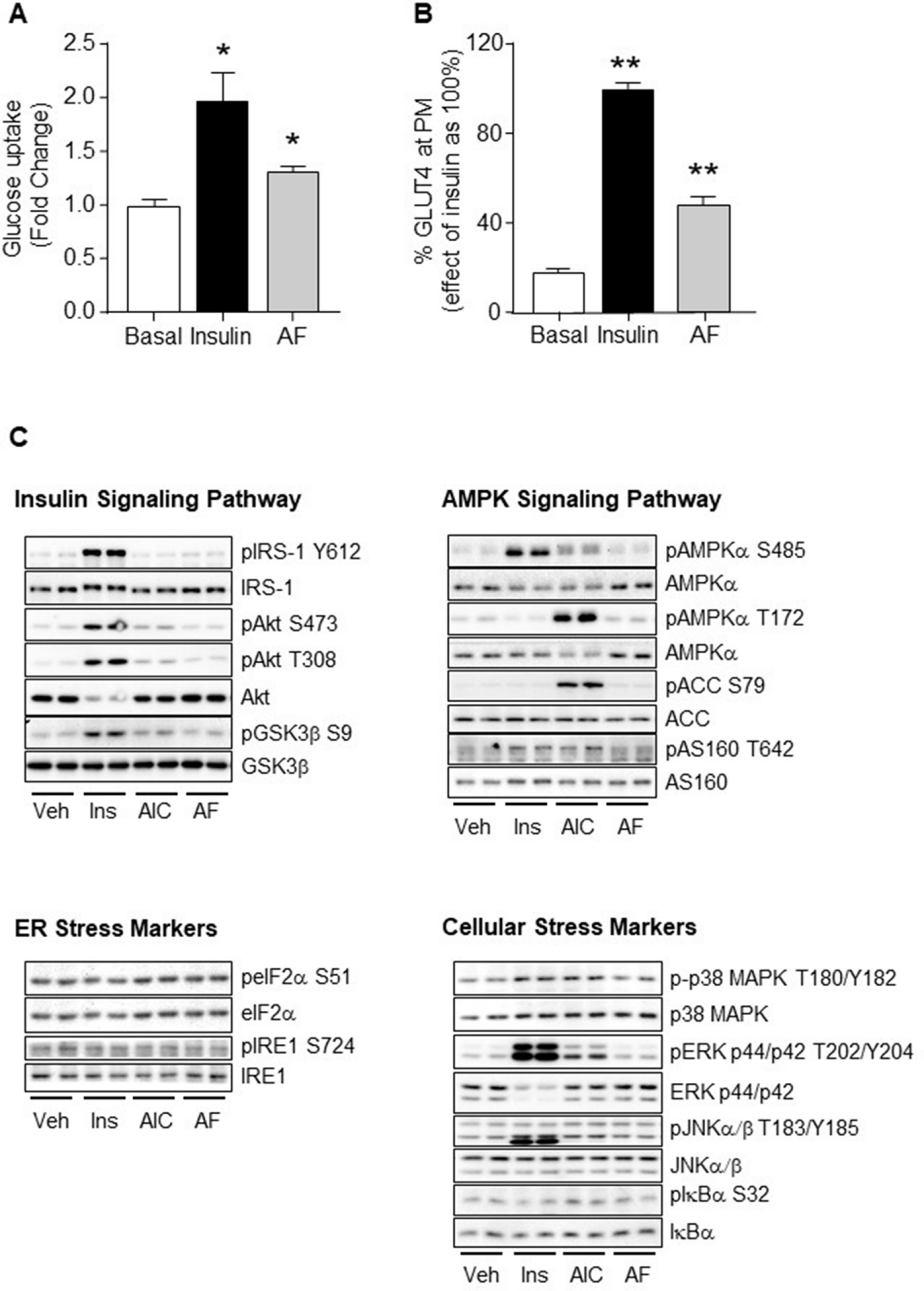
Effects of AF in L6 myotubes. **(A)** Differentiated L6 myotubes were serum starved for 18 h with inclusion of 10 μM AF for the final 1 h before washing out, the basal and insulin stimulated glucose uptake was then measured. The results were expressed as mean fold changes ± SEM over basal. **(B)** Differentiated L6 myotubes over-expressing HA-GLUT4 were serum starved for 18 h before addition of 10 μM AF for 1 h, followed by either basal (DMSO containing saline, final concentration of 0.2% DMSO), or 100 nM insulin for 30 min before the GLUT4 translocation from the cytosol to plasma membrane (PM) was measured. The results were quantified as a percentage of the maximum effect of insulin (100%) and expressed as means ± SEM. Three independent experiments were performed for each condition. *p < 0.05, **p < 0.01 vs. Basal. **(C)** Differentiated L6 myotubes were serum starved for 18 h before incubation with either vehicle (Veh), 100 nM insulin for 20 min, 2 mM AICAR for 50 min, or 10 μM AF for 1 h. Lysates were freeze-thawed before being cleared and subjected to Western Blot analysis for key regulators of the insulin, AMPK and stress signalling pathways. Three independent experiments were performed for each condition.

As AF improved glucose disposal in HF-fed mice, we next examined whether AF directly stimulates glucose uptake *via* GLUT4 translocation in L6 muscle cells. The results in **Figures 5A, B** showed that AF significantly increased glucose uptake (p < 0.05 vs Basal) and this was associated with increased translocation of GLUT4 to the plasma membrane (p < 0.01 vs Basal). These data indicate that AF can directly stimulate glucose uptake into muscle cells through increased GLUT4 translocation to the plasma membrane.

Given that AF acutely stimulated glucose uptake and GLUT4 translocation to the plasma membrane, we assessed the effects of AF on the insulin, AMPK and stress signalling cascades, the major mechanisms known to mediate GLUT4 translocation. As expected, both insulin and AICAR activated their specific signal pathways (**Figure 5C**). Insulin increased phosphorylation of IRS-1 (Tyr612), Akt (Ser473 and Thr308), GSK3β (Ser9), AMPKα (Ser485), AS160 (Thr642), p38 MAPK (Thr180), ERK (Thr202 and Tyr204), and JNKα/β (Thr183/Tyr185) while AICAR increased phosphorylation of ERK (Thr202 and Tyr204), AMPKα (Thr172 and partially Ser485), ACC (Ser79), AS160 (Thr642), and JNKα/β (Thr183/Tyr185). In comparison, AF did not cause direct phosphorylation of any of these signalling intermediates (**Figure 5C**). This indicates that AF is unlikely to stimulate glucose uptake and GLUT4 plasma membrane translocation in muscle cells mediated by the classical insulin, AMPK, ER, or cellular stress signalling cascades.

### Effects of AF on Whole-Body Energy Expenditure and Physical Activity

**Figure 6:**
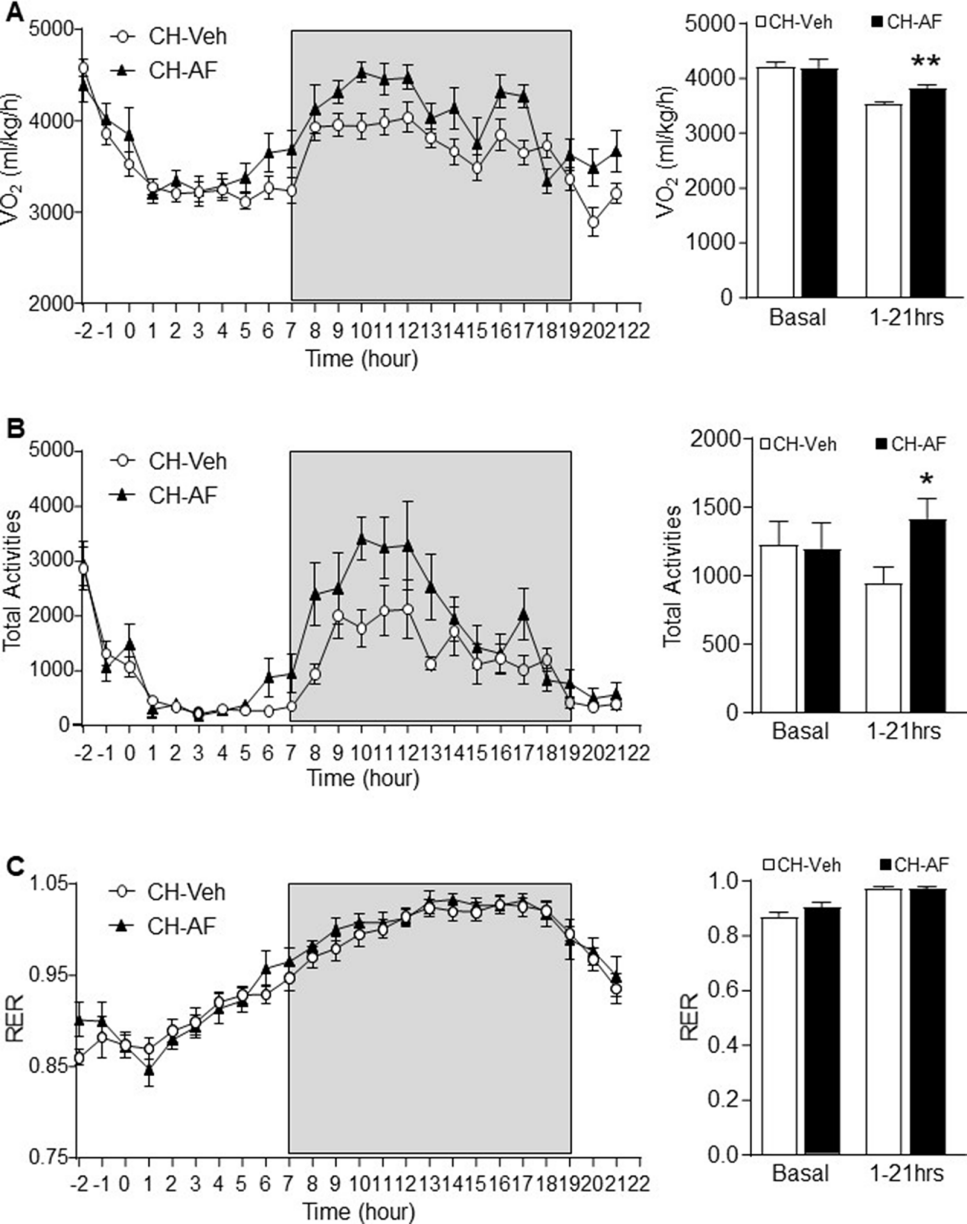
Effects of AF on whole-body energy expenditure and physical activity.A different batch of 12-week old CH-fed mice were acclimatised in a metabolic cage for 2 h followed by administration of vehicle (0.5% methylcellulose, CH-Veh group, white circle) or AF (100 mg/kg, CH-AF group, black triangle) by oral gavage. **(A)** Oxygen consumption rate (VO_2_), **(B)** total activities and **(C)** respiratory exchange ratio (RER) were monitored for 21 h after the drug administration (n = 13 for CH-Veh; 8 for CH-AF). Data are expressed as means ± SEM. *p < 0.05, **p < 0.01 vs. CH-Veh.

In a separate experiment, CH-fed mice (12-week old) were administrated with either vehicle (0.5% methylcellulose) or AF (100 mg/kg) to examine their whole-body energy expenditure and physical activities. AF significantly increased oxygen consumption (VO_2_) (**Figure 6A**) and total activities (**Figure 6B**) for at least 10 h after its administration, which is consistent with the data of reduced body weight gain, hepatic lipid accumulation, and adiposity. In addition, AF did not change the respiratory exchange ratio (RER) compared to CH mice (**Figure 6C**). These indicate that AF increased energy expenditure without using fatty acid as the predominant source for energy production.

## Discussion

AF is a small molecule that can be extracted from *Paeoniae radix* (Morinaga et al., 2013). It has a good oral absorption property and pharmacokinetics *in vivo* (Li et al., 2011). AF was reported to possess several attributes that might be beneficial for MetS-associated conditions (Hsu et al., 1997; Yang et al., 2004; Zhang et al., 2009; Juan et al., 2010; Zeng et al., 2012b). The present study further demonstrated its clear therapeutic effects *in vivo* for MetS and two major serious metabolic consequences, T2D and NAFLD/NASH, in three different mouse models that closely resemble features of MetS in humans. Our results indicate that the mechanism underlying these therapeutic effects is likely to be different from the current prospective anti-diabetic therapies, which involve the activation of the AMPK pathway, such as metformin (Cleasby et al., 2004), berberine (Lee et al., 2006), triterpenoids from bitter melon (Zeng et al., 2012a), and A-769662 (Hegarty et al., 2009). The present study suggests that AF may has a therapeutic potential for MetS and related diseases *via* promoting physical activity associated energy expenditure.

We firstly assessed the effects of AF on obesity and glucose tolerance in a HF-fed mouse model, because this model has been widely used for obesity, dyslipidaemia, and insulin resistance research (Turner et al., 2007; Tan et al., 2008). Our results showed that administration of AF significantly alleviated HF-induced obesity and associated glucose intolerance *in vivo*. As HF-fed mice do not develop overt T2D (Turner et al., 2007; Tan et al., 2008), a second model was employed for the evaluation of the efficacy of AF on typical hyperglycaemia. We next examined the efficacy of AF in a model of overt T2D induced by HF fed with multiple low doses of STZ (Zeng et al., 2012a). In this model of fully-developed T2D, AF clearly showed its efficacy to lower the hyperglycaemia. This effect is also consistent with the previous reports on the anti-hyperglycaemic effect of its analogue paeoniflorin (Hsu et al., 1997; Lau et al., 2007). Furthermore, we found that AF significantly enhanced the glucose disposal from the circulation during an ipITT. As skeletal muscle is mainly responsible for insulin-stimulated glucose disposal (DeFronzo and Tripathy, 2009), this anti-hyperglycaemic effect is possibly due to its effect to increase glucose uptake in this tissue.

MetS, together with persistent hyperglycaemia, is often associated with complications in various organs. An important goal for the treatment of T2D is to prevent its complications. Interestingly, one earlier study has reported the effect of AF to protect against diabetic kidney complication (Zhang et al., 2009). We considered it important to extent the study to examine the possible beneficial effects of AF for NAFLD/NASH, because there is currently no approved drug for this common but serious manifestation of MetS in the liver (Friedman et al., 2018). This experiment was conducted in chronic HF-HC fed mice, which has been increasingly recognized as a useful model representing the NAFLD/NASH in humans (Ioannou, 2016; Asgharpour et al., 2016). Indeed, we found that AF was able to reverse the increased liver triglyceride and cholesterol in HF-HC-fed mice, indicating its efficacy for hepatic steatosis (fatty liver). Furthermore, AF showed beneficial effects to lessen NASH-like phenotypes including reduced plasma level of AST (indicative of alleviated liver injury) and the expression level of TNFα mRNA in the liver (indicative of suppression of liver inflammation). These findings are reported for the first time, although it is currently not known whether these properties of AF involve pharmacological effects additional to the reduced obesity.

To further investigate the effects of AF on muscle metabolism, we used cultured L6 myotubes and showed that AF was capable to increase glucose uptake. Consistent with this result, AF increased the plasma membrane GLUT4 translocation suggesting that this is the means by which AF increases glucose uptake. As the insulin, AMPK an stress pathways are known to be the major mediators of GLUT4 translocation, we investigated the effects of AF on phosphorylation of key signalling intermediates in these pathways. Interestingly, we observed that AF did not have effect on any of the signalling intermediates. However, TBC1D1, a AMPK substrate, plays an important role in insulin-stimulated glucose transport of skeletal muscle (Cartee, 2015). It is worthwhile to examine the changes of TBC1D1 to exclude the AMPK activation in the future.

Previous studies showed that an AF analogue could increase GLUT4 translocation in adipocytes through the activation of the adenosine A1 receptor (AA1R) (Lai et al., 1998; Tang et al., 2003). Activation of AA1R by adenosine is associated with the phosphorylation of p38 MAPK, ERK, and JNK (Robinson and Dickenson, 2001; Ciccarelli et al., 2007; Migita et al., 2008; Xie et al., 2009). We examined the phosphorylation of these intermediates in response to AF, but we did not observe any change. Recently, there is emerging evidence that activation of AA1R can mediate GLUT4 translocation and glucose uptake *via* mTORC2-coupled influx of Ca^2+^ into cells independent of Akt (Sato et al., 2018). Ca^2+^-mediated GLUT4 endocytosis (Li et al., 2014) and glucose uptake (Witczak et al., 2007) can be independent of AMPK. Based on these reports, we speculate that AF may promote GLUT4 translocation and glucose uptake *via* the AA1R-mTORC2/Ca^2+^ mechanism.

At the whole-body level, we observed that AF promoted energy expenditure and increased physical activities in CH mice. This is interesting because administration of AF to experimental animals can increase the mobility time and movement frequency which are attributed to the increased expression of brain-derived neurotrophic factor (BDNF) in the hippocampus region (Wang et al., 2016). It has been reported that deletion of BDNF in the hippocampus suppresses both energy expenditure and physical activity (An et al., 2015) whereas pharmacological stimulation of BDNF increases both energy expenditure and physical activity in both CH- and HF-fed mice (Scabia et al., 2018). Based on these reports, we speculate that the therapeutic effects of AF on the MetS phenotypes may result from the increased physical activity-associated energy expenditure driven by its effects on the hippocampal BDNF. Interestingly adenosine receptor has also been suggested to be an upstream regulator of BDNF (Rodrigues et al., 2014). Whether AF-induced increases in physical activity and energy expenditure is driven by the adenosine-BDNF mechanism warrants further study.

TCM has been used in humans for many centuries for various diseases, which were described differently from the modern medicine. We have targeted this rich resource for the identification of new therapeutics or for the new application based on the new biological discoveries (Turner et al., 2016). Thus far, the identified berberine and triterpenoids exert the anti-diabetic properties with activation of the AMPK pathway albeit by different upstream mechanisms (Lee et al., 2006; Tan et al., 2008; Turner et al., 2008). It is clear that the therapeutic effects of AF on MetS are mediated by a different mechanism. Our findings, together with literature reports, appear to point to AA1R as a possible target of AF for its anti-MetS effects and this possible mechanism has not been reported for other TCM-derived therapeutics.

In conclusion, the present study in three mouse models within distinct phenotypes of the MetS demonstrated that AF exerts pharmacological properties to alleviate obesity, glucose/insulin intolerance, improve glycaemic control, and protect against the development of NASH. These beneficial effects of AF appear to be associated with its promotion of whole-body expenditure and glucose uptake in muscle. The findings from the present study provide a solid basis for that warrant further studies to repurpose the use of AF for MetS associated diseases *via* a mechanism distinct from other therapeutics derived from Chinese medicine.

## Data Availability Statement

The datasets generated for this study are available on request to the corresponding author.

## Ethics Statement

All animal experiments were approved by the Animal Ethics Committee of RMIT University (Approval No. #1208) in accordance with the guidelines issued by the National Health and Medical Research Council of Australia.

## Author Contributions

J-MY conceived the study and designed experiments with XZ and DL. XZ performed most of the experiments. X-YZ and SF participated in the experiments and tissue assays. J-MY, XZ, and DL analysed data and wrote the manuscript. JX and KZ provided advice, reagents and analytical tools to the study and critical comments on the manuscript.

## Funding

This work was supported by NHMC Program Grant of Australia (535921), National Natural Science Foundation of China (81870608) and Jiangmen Program for Innovative Research Team (China) (2018630100180019806).

## Conflict of Interest

The authors declare that the research was conducted in the absence of any commercial or financial relationships that could be construed as a potential conflict of interest.
